# Chinese Youths’ Physical Activity and Flourishing During COVID-19: The Mediating Role of Meaning in Life and Self-Efficacy

**DOI:** 10.3389/fpsyg.2022.867599

**Published:** 2022-05-18

**Authors:** Jun Zhou, Yongquan Huo

**Affiliations:** ^1^School of Psychology, Shaanxi Normal University, Xi’an, China; ^2^School of Physical Education, Shaanxi Xueqian Normal University, Xi’an, China

**Keywords:** physical activity, flourishing, mediation, COVID-19, meaning, self-efficacy

## Abstract

Physical activity has wide-ranging consequences for people’s physical, mental, and social health. Although the beneficial effects of physical activity on well-being were widely studied, how it promotes well-being remained unclear. The present study utilized the measure of physical activity rating scale (PARS-3), flourishing scale (FS), Chinese- meaning in life questionnaire (C-MLQ), and general self-efficacy scale (GSES) to examine the connection between physical activity and flourishing and the multiple mediation effects of meaning and self-efficacy with 827 Chinese undergraduates. The results indicated that (a) physical activity positively predicted flourishing; (b) meaning in life and self-efficacy played mediating roles in physical activity and flourishing, separately and jointly. Our findings revealed the mechanism of physical activity fostering flourishing, thereby providing an empirical basis for promoting health and flourishing, especially during COVID-19.

## Introduction

*Vita in motu. Life is in motion. (Voltaire, French thinker).*

The COVID-19 pandemic has made much impact on youths’ physical, mental, and social health. The unpredictability, uncertainty tolerance, and overloading of information during COVID-19 caused worries, fear, insecurity, and psychological stress (Exner-Cortens et al., 2021; Tetreault et al., 2021). The lockdown, social distancing, and home confinement brought about self-isolation and loneliness, resulting in reductions in life satisfaction and flourishing (Duong, 2021; Sürücü et al., 2021). Meanwhile, emerging research has found that physical activity exerted advantageous effects on physical and psychological well-being during the COVID-19 epidemic (Randall et al., 2021), but little is known about how people’s well-being benefits from their engagement in physical activity. Hence, it was of significance to reconsider the association between physical activity and flourishing and explore the underlying mechanism under the complicated condition of COVID-19 pandemic.

### Flourishing and Physical Activity

*Flourishing* is defined as the holistic well-being of physical and psycho-social levels, reflecting a better functioning in a broad range of one’s life (Keyes and Annas, 2009). Existing studies have found it is subject to the spread of COVID-19 and the subsequent adverse psychological effects (Elemo et al., 2021; Sürücü et al., 2021). *Physical activity* is conceptualized as body movements that contract skeletal muscles and expend energy. Substantial literature supports that physical activity is a positive protective factor for mental health and well-being across various age groups (Kekäläinen et al., 2020; Zheng et al., 2020).

Physical activity has been found to benefit well-being by effectively buffering the adverse impact of quarantines under the COVID-19 outbreak (Amatriain-Fernández et al., 2020). First, physical activity helps mitigate perceived worries, fear, anxiety, stress, and other negative feelings to maintain well-being. For example, López-Bueno et al. (2020) showed that physical activity is associated with a lower level of anxiety and bad mood. The study by Randall et al. (2021) shows that physical activity protects people from the suffering of perceived stress and depressive symptoms and positively predicts their psychological well-being. Second, physical activity potentially promotes personal psychological resources, including self-efficacy, to cope with the greatly changing situations. Camp et al. (2022) found that physically active people experienced more self-control and self-efficacy than inactive people, which helped their life function well.

### The Mediation of Meaning and Self-Efficacy

*Meaning* refers to finding and realizing things important or significant in people’s lives (Steger et al., 2006) and is considered as one of the basic motivations that reflect human existential value (Frankl, 1985). Positive psychologists claim that meaning is essential to a good life and lays the foundation for flourishing (Ryff and Singer, 2008). However, a great amount of research reported that people experienced decreased meaning during the COVID-19 pandemic (Karataş and Tagay, 2021; Karataş et al., 2021). The fluctuations in perceived meaning may be caused by fear of COVID-19, home isolation, and a sense of confinement, which can be primarily changed by engaging in physical activities (Yu et al., 2021). Bodily movements are conducive to direct attention from the outside world toward own body and the inside world, which contribute to finding important life goals and thus promote meaning and enhance flourishing.

*Self-efficacy* is developed as the confidence or belief that individuals are capable of realizing anticipated goals and achievements. From a developmental perspective, self-efficacy is an important psychological asset that can protect people from the threat of adversity and challenges, such as exposure to the stress situation of COVID-19 (Talsma et al., 2021; Yenen and Çarkit, 2021). Cataldi et al. (2021) found that students assigned to the physical exercise group received a significant increase in self-efficacy compared to students without bodily exercise, indicating that self-efficacy could develop from regular engagement in physical activities. Besides, existing studies supported that self-efficacy was a critical antecedent and determinant factor of well-being (Céspedes et al., 2021; Wang et al., 2021). In line with these findings, we observed that physical activity fosters self-efficacy and thus nourishes flourishing, so we assumed another path that physical activity would boost flourishing *via* the mediation of self-efficacy.

Theoretical and empirical evidence support that meaning and self-efficacy may individually and together mediate the connection between physical activity and flourishing. According to the risk and protective factor framework, meaning in life exerts a protective influence on self-efficacy under the risk of excessive stress (Masten, 2001). Moreover, Yuen and Datu (2021) suggest that meaning in life positively correlated with general self-efficacy and specific self-efficacy (Yuen and Datu, 2021). Self-efficacy has also been found to mediate the interrelation of meaning and psychological well-being (Krok and Gerymski, 2019; Czyżowska and Gurba, 2021). Both, perceived meaning and self-efficacy, are malleable psychological resources that directly or indirectly boost flourishing, which can be activated, maintained, and promoted by physical activities. Hence, the present study also tested the serial mediation effect of meaning and self-efficacy in the relationship between physical activity and flourishing.

### Gender Differences

It also should be noted that gender differences existed in some of our main variables, including physical activity, self-efficacy, flourishing, and the response to stressful COVID-19. It is well-documented that boys reported significantly more engagement in physical activity and higher physical activity self-efficacy than girls, and girls experienced more barriers to exercise participation (Chen et al., 2019; Rosselli et al., 2020). Also, Sürücü et al. (2021) showed that females flourishing are more vulnerable to COVID-19 fear than males. Thus, the present study takes gender as a covariant in the mediation model to avoid the confusion induced by gender differences.

### The Present Study

Under the complex and volatile situation of COVID-19, our primary purpose was to probe the positive cognitive and behavioral factors that may help people cope with the possible adverse psychological problems or even vicarious traumatization. The current study investigated the advantageous effects of physical activity on flourishing *via* the mediation effects of two kinds of psychological resources, meaning and self-efficacy. Four hypotheses were proposed:

H1: Physical activity positively predicts flourishing.
H2: Meaning mediates the connection between physical activity and flourishing.
H3: Self-efficacy mediates the connection between physical activity and flourishing.
H4: Meaning and self-efficacy mediate the connection between physical activity and flourishing together.

## Materials and Methods

### Participants and Procedure

A total of 873 students from 3 universities in Xi’an, a provincial capital city in northwest China, participated in an online survey. After asking for informed consent, we sent the website of Questionnaire Star to the students, and they voluntarily and anonymously completed four well-validated questionnaires and related information (major, gender, and age). The data of 46 participants were dropped out due to reasons such as failing attention-check questions (e.g., “please choose strongly disagree to this item”), too short answer time (less than 60 s), and similar response styles (e.g., selecting the same answer in all items). The final sample consisted of 827 participants (recovery rate = 94.73%, 59.7% females; Mean (*M*_*age*_) = 19.23, Standard deviation (*SD*_*age*_) = 1.39). According to the analysis in G-power, the sample size was sufficient to detect a medium-sized effect (*r* = 0.30, α = 0.05, 1 – β = 0.80).

### Measure

#### Physical Activity

The physical activity rating scale (PARS-3), designed by Hashimoto (1990) and translated and revised by Liang and Liu (1994), was a 3-item measure assessing the level of physical activity. Participants were asked to rate the frequency, duration, and intensity of their bodily movements from 1 to 5. The total score for physical activities was the product of the scores on frequency, duration (minus 1), and intensity. The scale has good psychometric properties and has been widely used for young adults in Chinese culture (Chen and Ji, 2006; Zheng et al., 2020). The test–retest reliability in Liang and Liu (1994)’s study was 0.82. The Cronbach alpha coefficient of PARS-3 in this study was 0.65.

#### Flourishing

The 8-item flourishing scale (FS) was used for assessing the level of flourishing (Diener et al., 2010). Participants were asked to rate each item on a Likert scale (1-7) anchored from “strongly disagree” to “strongly agree.” The FS shows good psychometric properties and has been validated in various countries and populations (Kazak et al., 2021). The Cronbach alpha coefficient of FS in this study was 0.93. The measurement performed a good fit: χ^2^/*df* = 4.26, goodness of fit index (GFI) = 0.982, comparative fit index (CFI) = 0.990, Tucker-Lewis index (TLI) = 0.980, root mean squared error of approximation (RMSEA) = 0.063, and standardized root mean square residual (SRMR) = 0.019.

#### Meaning

We employed the meaning in life questionnaire (MLQ), developed by Steger et al. (2006), to quantify how students perceive their life as purposeful or meaningful (POM). It has ten sentences that describe the perception of important life purpose or searching for life purpose, scored on a Likert scale (1-7). The MLQ has good psychometric properties and has been validated in various countries and populations (Elizabeth and Chang, 2021). The POM sub-scale was applied to this study and yielded an alpha of 0.79. The measurement performed a good fit: χ^2^/*df* = 2.52, GFI = 0.994, CFI = 0.996, TLI = 0.993, RMSEA = 0.043, and SRMR = 0.015.

#### Self-Efficacy

The present study employed the general self-efficacy scale (GSES) to quantify students’ perceived self-efficacy at the broadest level (Schwarzer and Jerusalem, 1995; Wang et al., 2001). The 10-item scale was scored on a Likert scale from 1 to 4 (1 = not at all true, 4 = exactly true). Individuals with higher scores perceive a high level of self-efficacy. Meanwhile, the psychometric properties of GSES have been examined in various countries (Sekerdej and Szwed, 2021). Its Cronbach’s alpha coefficient was 0.87 in the current study. The measurement performed a good fit: χ^2^/*df* = 3.58, GFI = 0.975, CFI = 0.974, TLI = 0.960, RMSEA = 0.056, and SRMR = 0.030.

### Data Analysis

SPSS 25.0 and PROCESS MACRO V 3.4 were utilized to analyze data as follows:

(1) The means, standard deviations, and bivariate correlation coefficients of primary variables were calculated in SPSS 25.0.
(2) The independent *t*-test was conducted to examine the differences between male and female students in their physical activity engagement because previous studies have found gender differences in this variable.
(3) A multiple mediation model was established to investigate the single and serial mediation effects of meaning and self-efficacy on the connection between physical activity and flourishing using PROCESS MACRO V 3.4.

## Results

### Descriptive Statistics and Correlation Analysis

The results are presented in Table 1, including means, *SD*, and correlation coefficients of primary variables. Physical activity, flourishing, meaning, and self-efficacy were significantly and positively correlated with each other (*ps* < 0.001).

**TABLE 1:** Means, SD, and correlation coefficients of major variables.

	*M*	SD	1	2	3	4
1. Physical activity	9.59	8.93	–			
2. Meaning in life	5.49	0.98	0.13***	–		
3. Self-efficacy	2.89	0.40	0.23***	0.31***	–	
4. Flourishing	5.66	0.87	0.11**	0.64***	0.55***	–

**p < 0.01 and ***p < 0.001.

Before examining the effect of mediators, an independent *t*-test was applied to analyze the gender differences. As shown in Table 2, the difference between female and male participants was significant (*t*_(825)_ = 19.37, *p* < 0.001, 95% C.I. = [9.14, 11.20], *r* = 0.55). Male students reported more physical activities than females. For this reason, we controlled gender in the following mediation analysis.

**TABLE 2:** Difference between male and female students engaging in physical activity.

	*M*	SD	*t*	Cohen’s *d*
Female students (*n* = 494)	5.49	6.09	19.37***	1.32
Male students (*n* = 333)	15.66	9.01		

***p < 0.001.

### Mediation Effect Analysis

PROCESS macro was used to test the multiple mediating effects of meaning and self-efficacy in the connection between physical activity and flourishing, by selecting model 6 and setting the sample size as 5,000, physical activity as the independent variable, flourishing as the dependent variable, perceived meaning and self-efficacy as two mediators, and gender as a covariate. The regression analysis results (see Table 3) showed that students’ physical activity positively predicted their perceived meaning in life (β = 0.121, *p* = 0.004), self-efficacy (β = 0.126, *p* = 0.001), and flourishing (β = 0.127, *p* = 0.003); meaning predicted self-efficacy (β = 0.282, *p* < 0.001) and both of them predicted flourishing (β_*meaning*_ = 0.519, *p* < 0.001; β_*self–efficacy*_ = 0.406, *p* < 0.001). The hypothesis H1 was proved.

**TABLE 3:** Model fit indices and standardized regression coefficients.

Model		Model fit indice			Standardized coefficient	
Outcome	Predictor	*R*	*R* ^2^	*F*	β	*t*
Meaning	Gender	0.133	0.018	7.453	0.021	0.418
	Physical activity				0.121	2.896**
Self-efficacy	Gender	0.372	0.138	43.964	0.111	2.847**
	Physical activity				0.126	3.198**
	Meaning				0.282	8.638***
Flourishing	Gender	0.741	0.549	250.065	−0.082	−2.897**
	Physical activity				−0.001	−0.018
	Meaning				0.519	21.006***
	Self-efficacy				0.406	16.089***

**p < 0.01 and ***p < 0.001.

**FIGURE 1:**
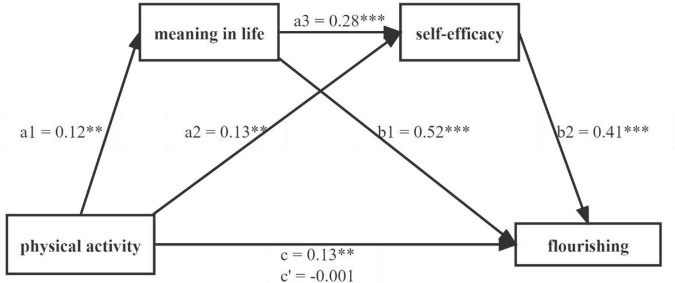
The multiple mediation effect of meaning in life and self-efficacy (*N* = 827). a1, a2, a3, b1, b2, c, c’ are standardized coefficients of paths; ^**^p < 0.01, ^***^p < 0.001.

The bias-corrected percentile bootstrap method was applied to examine the indirect effects, and the results showed that three indirect paths were significant (see Figure 1 and Table 4). Specifically, neither the 95% credit interval of the mediating effect of meaning in life overlapped zero (from 0.016 to 0.110) nor the 95% credit interval of the mediating effect of self-efficacy included 0 (from 0.018 to 0.086). Supporting the hypotheses H2 and H3, meaning and self-efficacy individually mediate the relationship between physical activity and flourishing. Notably, the serial mediation of meaning in life and self-efficacy was significant because the 95% credit interval was from 0.003 to 0.028, which did not overlap with zero. The hypothesis H4 was proved, indicating that flourishing is promoted by physical activity through the sequential path of meaning and self-efficacy.

**TABLE 4:** The multiple mediation effects of meaning in life and self-efficacy.

Path	Indirect effect	Boot SE	95% CI		Relative indirect effect%
Total	0.127	0.032	0.066	0.190	
Ind1	0.063	0.024	0.016	0.110	49.61%
Ind2	0.051	0.017	0.018	0.086	40.16%
Ind3	0.014	0.006	0.003	0.028	11.02%

*Ind1: physical activity→meaning in life→flourishing;*

*Ind2: physical activity→self-efficacy→flourishing;*

*Ind3: physical activity→meaning in life→self-efficacy→flourishing.*

## Discussion

There’s no denying how COVID-19 has created many differences in our lives, and we have no idea of its end yet. To discover the contributing factors that facilitate people to fight the crisis, the present study tested the relationship between youths’ physical activity and flourishing and uncovered the inner mechanism of how flourishing benefits from physical activity by conducting a cross-sectional study among Chinese undergraduates. The results suggested that physical activity positively predicted flourishing *via* the individual and joint mediation effects of meaning and self-efficacy. It was of great significance to examine the underlying pathways from physical activity to flourishing for developing practical solutions that protect people from the stressful situation of COVID-19.

The first finding was that more engagement in physical activities predicted a higher level of flourishing, implying the buffering effect of physical activity on flourishing under the pressure of COVID-19. Much research proves that the COVID-19 pandemic impairs people’s physical and mental health and well-being, triggering tension, panic, fear, anxiety, and depression (Duong, 2021; Elemo et al., 2021; Exner-Cortens et al., 2021; Zammitti et al., 2021). Even if one did not get COVID-19, one might experience vicarious traumatization (Xu and Liu, 2021). Physical activity is a protective buffering factor in facing this complex situation. It is helpful to divert our attention from the overloading information on COVID-19, relax our bodies, and ease our tension, thereby obviating the possibility of emotional problems (López-Bueno et al., 2020; Randall et al., 2021). Our study supported the advantageous effects of physical activity on mental health and well-being.

Importantly, our second finding was that flourishing could benefit from physical activity from three mediating paths: meaning, self-efficacy, and together. Based on the embodied cognition theory (Shapiro, 2019), the body is the prerequisite for developing the mind, and action and experience of the body shape and boost the development of cognition and affection and further leverage over well-being. González-Hernández et al. (2019) advocated that the development of psychological resources should be taken into account for teenagers’ perfect way of doing sports. Supporting this point, our study showed that increased engagement in physical activity could help one focus on the self, physically and psychologically, which enriched psychological resources like promoted perceived meaning (Hooker and Masters, 2016) and self-efficacy (Suorsa et al., 2016), and could also facilitate recovery from the depletion of psychological resources (Hoover et al., 2021). In addition, our findings also approve the theoretical viewpoint that meaning and self-efficacy are two crucial psychological assets to deal with the stressful situation of the COVID-19 pandemic (Cataldi et al., 2021; Talsma et al., 2021; Yu et al., 2021).

The current research has revealed the psychological mechanism of how individuals benefit from physical efforts and vital psychological resources. However, several limitations need to be considered. First, this study unveiled the mediating role of meaning and self-efficacy, but its effect size was small to medium. There might be other inner mediators or moderators in the link between physical activity and flourishing that should be explored in the future, like gender (Sürücü et al., 2021). Second, a cross-sectional design was applied in our work, and a longitudinal study could be used in future work, for it might be more helpful to probe the contributing factors and causal relations. Third, we evaluated the total number of participants engaging in physical activity but did not include the types of physical activity, thereby limiting our findings in providing more specific recommendations for developing practical strategies to promote psychological resources and enhance flourishing. Fourth, a recent study shows a potential reciprocal relationship between physical activity and flourishing (O’Rourke et al., 2021), indicating the bidirectional relation needs to be considered in future research.

The COVID-19 epidemic has brought unmeasurable negative effects to the economy and society, and it is still looming large in our lives, but we can fight against it with our own positive psychological strengths, such as meaning and self-efficacy. Physical activity is an effective way to build these psychological strengths and shield us from bad influences, whether now or before the COVID-19 pandemic. Our study validated the positive effects of body movement on flourishing, and examined the mediation role of meaning and self-efficacy on the positive relation of physical activity and flourishing, thereby providing an empirical basis for an embodied way to launch life meaning education and improve flourishing during COVID-19.

## Data Availability Statement

The original contributions presented in the study are included in the article/Supplementary Material, further inquiries can be directed to the corresponding author.

## Ethics Statement

The studies involving human participants were reviewed and approved by the Ethics Committee of Shaanxi Normal University. The patients/participants provided their written informed consent to participate in this study.

## Author Contributions

YH instructed JZ to conduct this study, including the initial idea, language polishing, and method. JZ was a doctoral candidate of YH and collected, analyzed the data, wrote the manuscript, made tables, and drew figures. Both authors contributed to the article and approved the submitted version.

## Conflict of Interest

The authors declare that the research was conducted in the absence of any commercial or financial relationships that could be construed as a potential conflict of interest.

## Publisher’s Note

All claims expressed in this article are solely those of the authors and do not necessarily represent those of their affiliated organizations, or those of the publisher, the editors and the reviewers. Any product that may be evaluated in this article, or claim that may be made by its manufacturer, is not guaranteed or endorsed by the publisher.
